# Biological Cover Mitigates Disruption of the Dermal Structure in Mechanically Expanded Skin in a Porcine Model

**DOI:** 10.3390/ijms232113091

**Published:** 2022-10-28

**Authors:** Joanna K. Ledwon, Sarah A. Applebaum, Bianka Progri, Oveyaa Vignesh, Kristof S. Gutowski, Alec B. Chang, Adrian B. Tepole, Arun K. Gosain

**Affiliations:** 1 Department of Surgery, Northwestern University Feinberg School of Medicine, Chicago, IL 60611, USA; 2 Department of Plastic and Reconstructive Surgery, Stanley Manne Children’s Research Institute, Ann and Robert H. Lurie Children’s Hospital of Chicago, Chicago, IL 60611, USA; 3 Department of Mechanical Engineering, Purdue University, West Lafayette, IN 47907, USA

**Keywords:** tissue expansion, acellular dermal matrix, mechanical forces, macrophages, dermis, collagen deposition, capsule, radiation

## Abstract

Tissue expansion is an integral procedure of the vast majority of breast reconstruction and has a significant impact on the final clinical outcomes. Therefore, technological advances leading to a fewer number of unfavorable outcomes and a decrease in complication rates are imperative. In this study, using a porcine model, we investigated an effect of acellular dermal matrix (ADM) used as a tissue expander cover on the dermal changes induced by mechanical forces during tissue expansion. After 14 days of expansion, skin samples were collected from one animal, while the second animal underwent radiation, and tissue was collected 8 weeks later. Tissue expanded without the use of ADM and unexpanded skin served as the controls. Collected skin biopsies were used for histological and immunohistochemical evaluation, and for gene expression analysis. We revealed that the biological cover incorporation into host tissue is facilitated by macrophages without inducing a broad inflammatory response. The utilization of ADM mitigated disruption in the dermal structure, excessive collagen deposition, and capsule formation in non-irradiated expanded skin. The protective effect was not fully maintained in irradiated skin. These results demonstrate that tissue expansion might be improved by using the tissue expander cover.

## 1. Introduction

Acellular dermal matrix (ADM) is commonly used in breast reconstruction to provide support to the tissue expander or implant, prevent excessive pressure on mastectomy flaps, and improve aesthetic outcomes by defining the inframammary fold [1]. ADM proponents report enhanced control of the mastectomy pocket, leading to more optimal prosthetic positioning and improved cosmesis [2,3]. Regarding safety, a multicenter prospective study concluded that ADM use in immediate expander/implant breast reconstruction does not increase risk of complications in the general population [4], however it might confer a higher risk of major complications in patients with a BMI above 30 [5]. On a molecular level, it has been shown that ADM might decrease capsule formation [6,7,8] and limit the extent of radiation-induced soft tissue injury [9,10] in oncologic settings that require postmastectomy radiation therapy. However, these findings are limited to prospective observational studies with small sample sizes [11] and absence of a control group [12]. Furthermore, the potential benefits of utilizing ADM as a cover during tissue expansion have not been verified. Therefore, there is a need to evaluate the impact of ADM on skin growth in tissue expansion in a controlled setting to verify its potential therapeutic effect and provide an evidence-based guidance for the surgeons. 

Here, using a porcine model we investigated how a biological cover affects host immune response and the dermal changes induced by mechanical forces during tissue expansion. We evaluated the activity of macrophages and the expression of inflammatory markers in expanded skin. We explored changes in collagen fibers’ organization and deposition, and evaluated capsule formation induced by mechanical forces. These data may help to modernize the original technique by optimizing existing products and/or designing novel materials for safe and successful tissue expansion.

## 2. Results

### 2.1. Macrophages Facilitate Integration of a Biological Cover with Host Tissue 

**Figure 1:**
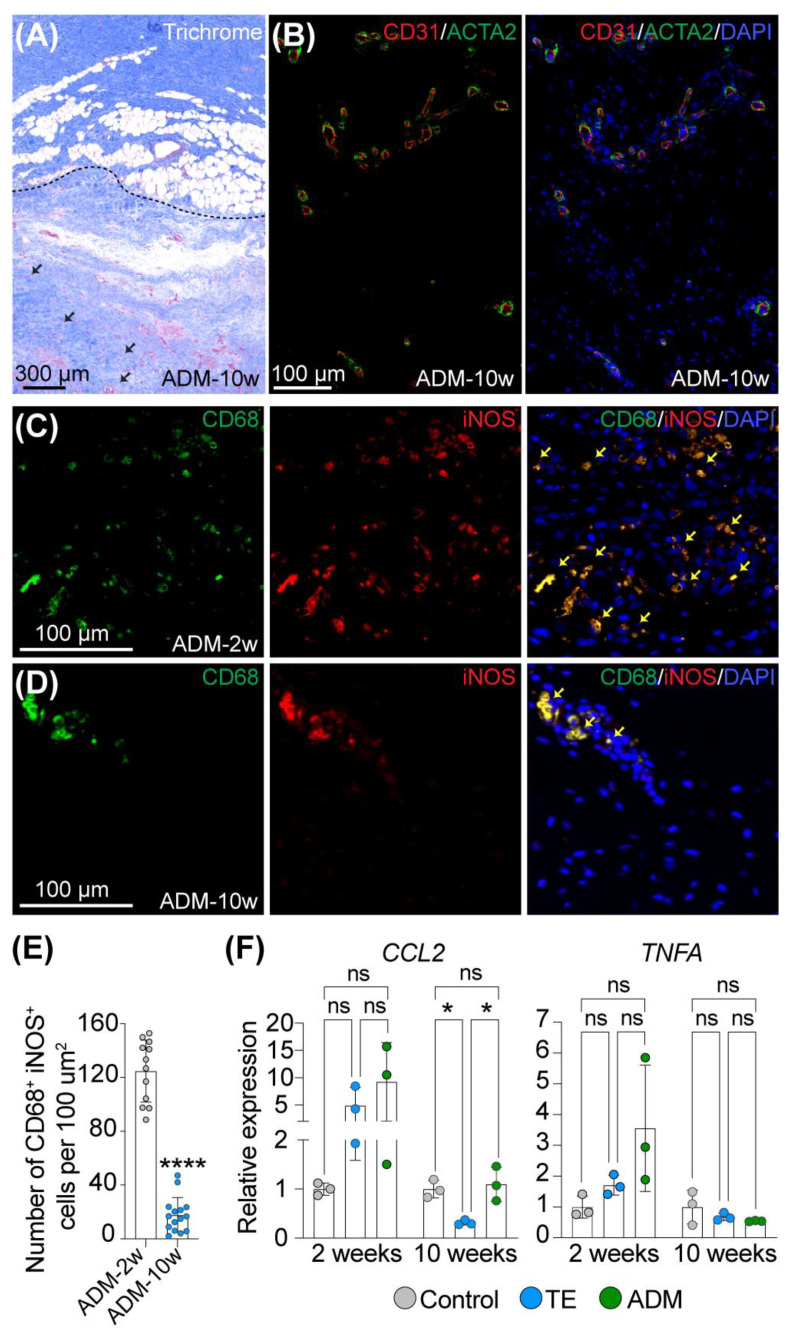
**Macrophages are temporally recruited to participate in incorporation of the biological cover into host tissue during expansion.** Representative images of (**A**) Trichrome staining of ADM attached to host tissue and (**B**) double immunofluorescence staining of CD31 and ACTA2 of ADM at 10 weeks of expansion. Dashed line and black arrows in (**A**) mark attachment of ADM to host tissue and cells infiltrating ADM, respectively. (**C**,**D**) Representative images and (**E**) quantitative analysis of double immunofluorescence staining of CD68 and iNOS of ADM attached to host tissue at (**C**) 2 weeks and (**D**) 10 weeks of expansion. The yellow arrows indicate double positive CD68^+^iNOS^+^ cells. In (**A**) slides were counterstained with hematoxylin and in (**B**–**D**) slides were counterstained with DAPI to mark nuclei (blue stain). (**F**) Relative expression of pro-inflammatory markers in control (Ctr) and expanded skin (TE and ADM model) assessed by qRT-PCR at 2 and 10 weeks of expansion compared to corresponding unexpanded control. The values were normalized to the geometric mean calculated for 2 reference genes: *RLPL0* and *RPS27A* and are presented as the average for 3 biopsies. Magnification in (**A**) 2×, in (**B**) 10×, in (**C**,**D**) 20×. Error bars represent SD. Statistical significance calculated in (**E**) with unpaired Student *t*-test and in (**F**) with one-way ANOVA with Bonferroni correction for multiple comparison is shown as * *p*-value < 0.01, **** *p*-value ≤ 0.0001, ns—not statistically significant.

Histological analysis of skin biopsies collected at 2 and 10 weeks of expansion showed successful incorporation of ADM into host tissue (Figure 1A). ADM was infiltrated by cells from neighboring tissue, as indicated by the presence of nuclei in the previously decellularized ADM (Figure 1A, black arrows). IF staining of CD31 and ACTA2, markers of endothelial cells and vascular smooth muscle cells, respectively, revealed the presence of CD31^+^ cells surrounded by ACTA2^+^ cells in ADM, indicating the ingrowth of newly formed blood vessels in ADM (Figure 1B). 

To examine the inflammatory response of host tissue to ADM, we performed double IF staining of CD68 and iNOS, which are expressed by inflammatory cells, specifically macrophages type M1. At week 2 of expansion, we observed a large number of CD68^+^iNOS^+^ cells residing in the area where ADM was attached to the host tissue (Figure 1C), whereas at week 10 of expansion, the number of CD68^+^iNOS^+^ cells significantly diminished (FC = 0.06, *p*-value < 0.0001) (Figure 1D,E). To further explore changes in the host immune response following tissue expansion with and without a biological cover, we evaluated the expression of inflammatory markers, *CCL2* (C-C Motif Chemokine Ligand 2) and *TNFA* (Tumor Necrosis Factor Alpha) in skin biopsies surrounding the tissue expanders at weeks 2 and 10 of expansion (Figure 1F). Interestingly, we did not detect a significant increase in *CCL2* and *TNFA* expression at any tested timepoint for any condition. However, we noticed a trend that samples collected at week 2 of expansion from the ADM model had consistently higher expression of these genes compared to other conditions, even though the TE samples were also slightly upregulated. Furthermore, at week 10 of expansion, the TE model showed a slight decrease in *CCL2* expression compared to control skin (FC = 0.31, adj. *p*-value = 0.0323) and ADM samples (FC = 0.22, adj. *p*-value = 0.0175). Overall, these results indicate that tissue expansion itself might affect and induce a mild host immune response, and use of a biological cover may slightly aggravate its local activity. Most importantly, the proinflammatory response observed in both tested tissue expansion models diminished completely over time. Simultaneously, a negligible number of macrophages was detected in the dermis of the control and expanded skin at both tested timepoints, suggesting that ADM does not induce a broad inflammatory response and its incorporation into host tissue is driven by macrophages. 

### 2.2. Biological Cover Diminishes Structural Changes in the Dermis Induced by Tissue Expansion

**Figure 2:**
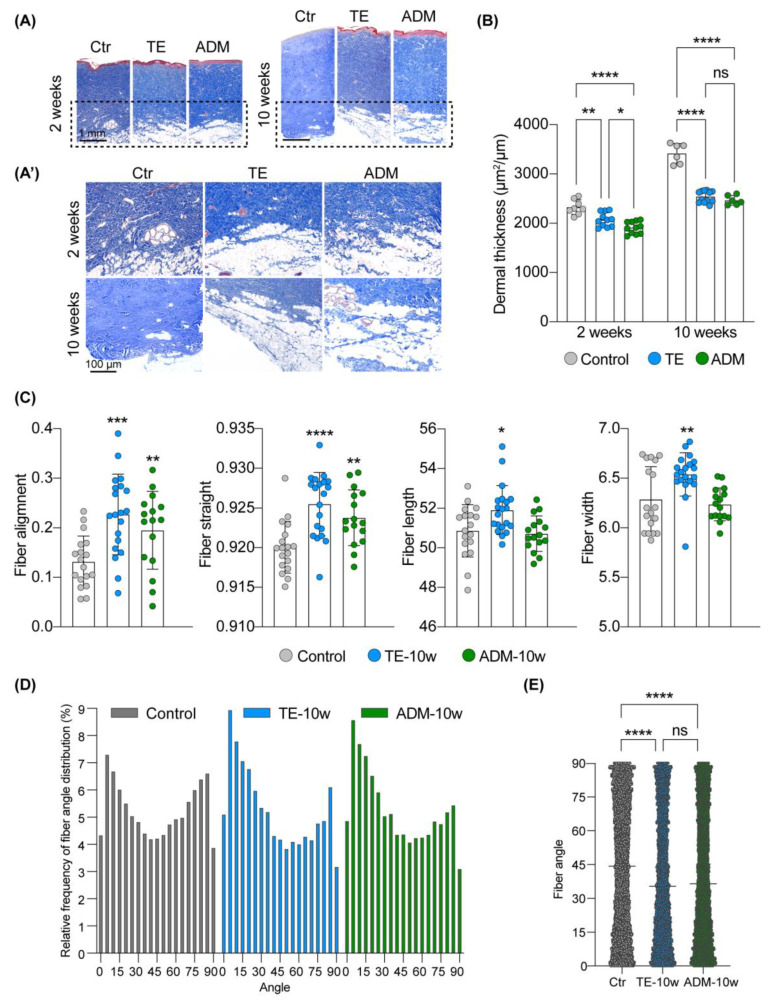
**Biological cover limits structural changes in the dermis caused by mechanical stretching.** (**A**,**A’**) Representative images and (**B**) quantitative analysis of the dermal thickness of control (Ctr) and expanded skin (TE and ADM model) stained with trichrome at 2 and 10 weeks of expansion. Panel (**A’**) presents the magnified views of the area marked in panel (**A**) (2× magnification). (**C**) Quantitative analysis of fiber organization in control (Ctr) and expanded skin (TE and ADM model) at 10 weeks of expansion on slides stained and analyzed with Picrosirius red and CT-FIRE, respectively. The values present the medians for each analyzed image and error bars represent SD. (**D**) Histogram and (**E**) bar graph of fiber angle distribution in the dermis of control (Ctr) and expanded skin (TE and ADM model) at 10 weeks of expansion, evaluated on Picrosirius red stained slides and analyzed with CT-FIRE software. The values present the averages for 9 images (3 images per biopsy) for each tested condition. Error bars represent SD. Statistical significance calculated in (**B**,**E**) with ordinary one-way ANOVA with Bonferroni correction for multiple comparisons and in (**C**) with unpaired Student *t*-test is shown as * *p*-value < 0.05, ** *p*-value ≤ 0.01, *** *p*-value ≤ 0.001, **** *p*-value ≤ 0.0001, ns—not statistically significant.

The highest magnitude of forces applied by the tissue expander is delivered to the dermis, the largest component of the skin. Therefore, we performed a robust histological evaluation of the dermis, including structural changes in collagen fibers’ organization, to assess the potential of the tissue expander cover in maintaining dermal homeostasis during expansion. At week 2 of expansion, we observed a decrease by 11% (adj. *p*-value = 0.0014) in the dermal thickness of expanded skin compared to unexpanded control skin (Figure 2A,B). When the tissue expander was wrapped in ADM the decline in dermal thickness was even more pronounced, resulting in an additional 7% decrease (adj. *p*-value = 0.0322) compared to the TE model, and 18% decrease (adj. *p*-value < 0.0001) compared to unexpanded control skin (Figure 2B). The decline in dermal thickness in expanded skin was even more pronounced at week 10 of expansion, representing a 26% (adj. *p*-value < 0.0001) and 29% (adj. *p*-value < 0.0001) decline in the TE and ADM models, respectively (Figure 2A,B). The difference between the TE and ADM model was no longer statistically significant (adj. *p*-value > 0.9999), indicating that the decline in dermal thickness caused by the use of ADM normalizes over time and does not progress more than in skin expanded without ADM.

To further explore the effect of the biological cover on structural changes in the dermis, we performed selective staining of collagen fibers using Picrosirius red staining on tissue collected at week 10 of expansion (Figure 2C–E). This histological staining paired with confocal imaging and the CT-FIRE software allowed for quantitative analysis of fiber orientation and properties. We determined that the tissue expander wrapped in the biological cover induces less pronounced morphological changes in the dermis during expansion than the tissue expander without a cover. Properties of collagen fibers that were significantly affected in the TE model compared to control, but not in the ADM model, included fiber length and width, indicating that the biological cover might act as a buffer to reduce disturbance in dermal homeostasis. Samples collected from the TE model had 2.1% more elongated (*p*-value = 0.0161) and 4% wider fibers (*p*-value = 0.0082). Simultaneously, expanded skin, regardless of the tissue expansion protocol, was characterized by an increase in fiber alignment (FC ≥ 1.48, *p*-value ≤ 0.0085) and straightness (FC ≥ 1.004, *p*-value ≤ 0.0032). Moreover, while in control samples we observed a more even distribution of horizontal (defined as fibers H = 0°–45°) and vertical fibers (defined as fibers V = 45°–90°), in expanded skin, fibers oriented horizontally were detected at least 1.32 times more frequently (Figure 2D,E). This phenomenon was revealed in both tissue expansion models. All together, these results indicate that during tissue expansion collagen fibers change their organization to adapt to external forces and preserve tissue integrity. However, tissue expansion with the use of a biological cover leads to less organizational and structural changes in dermal collagen. 

### 2.3. Biological Cover Prevents Excessive Collagen Deposition in Expanded Non-Irradiated Skin

**Figure 3:**
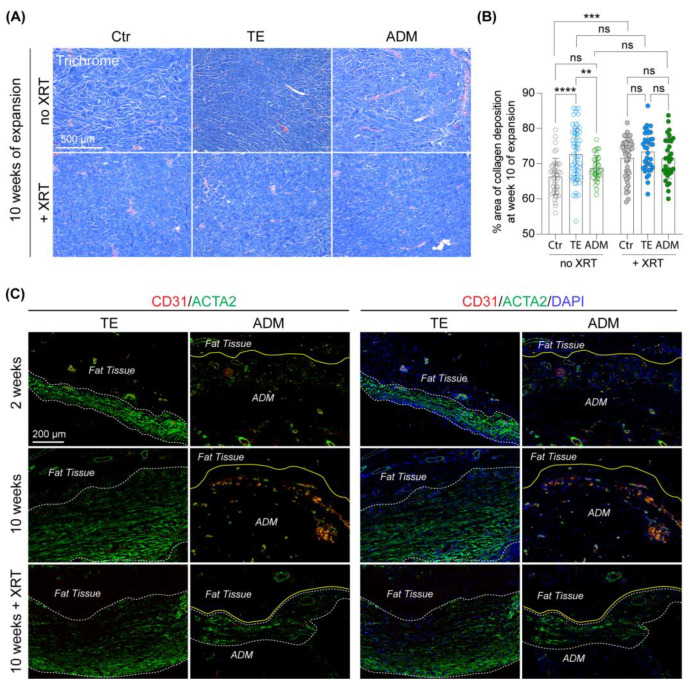
**Biological cover prevents excessive collagen deposition and capsule formation in expanded non-irradiated skin.** (**A**) Representative images and (**B**) quantitative analysis of trichrome staining of non-irradiated (no XRT) and irradiated (+XRT) skin samples at 10 weeks of expansion. Ctr, unexpanded control skin; TE, skin expanded without a biological cover; ADM, skin expanded with a biological cover. Magnification 10×. Scale bars 500 μm. Error bars represent SD. Statistical significance calculated with ordinary one-way ANOVA with Bonferroni correction for multiple comparisons is shown as ** *p*-value ≤ 0.01, *** *p*-value ≤ 0.001, **** *p*-value ≤ 0.0001, ns — not statistically significant. (**C**) Representative images of double immunofluorescence staining of CD31 and ACTA2 on tissue that was in close proximity to the tissue expander alone (TE model) or covered with a biological matrix (ADM model). Tissue samples were collected from non-irradiated skin at 2 and 10 weeks of expansion, and from irradiated skin at 10 weeks of expansion. White dashed lines outline the capsule formed above the tissue expander. Yellow lines mark the region where ADM is attached to host tissue.

The biomechanical properties of skin are largely dictated by collagen organization and density [13]. To compare changes in collagen deposition in skin samples expanded without (TE model) and with a biological cover (ADM model) we performed Trichrome staining on non-irradiated and irradiated skin biopsies collected at week 10 of expansion (Figure 3A,B). Radiation was administered 2 weeks after initiating expansion and tissue samples were collected 8 weeks later, allowing us to observe the microstructural changes and late radiation effects. The quantitative analysis with Fiji ImageJ software on trichrome stained non-irradiated skin samples revealed a significant increase in collagen deposition (FC = 9.6%, adj. *p*-value < 0.0001) when skin was expanded without a biological cover (Figure 3A,B). In contrast, skin samples expanded by the tissue expander wrapped in ADM showed more uniform collagen distribution, without excessive local collagen deposition, unlike that which was observed in skin samples harvested from the TE model. The average collagen deposition observed in the ADM model was not significantly changed compared to unexpanded control skin, but it was 5.4% (adj. *p*-value = 0.0197) lower than average collagen deposition in the TE model. These results indicate that use of ADM during tissue expansion might prevent excessive collagen deposition induced by mechanical forces applied by the tissue expander. 

One of the side effects of radiation is increased collagen deposition in irradiated soft tissue due to a premature differentiation of fibroblasts into mature collagen-forming fibrocytes [14]. As expected, we observed a significant increase in collagen deposition in unexpanded irradiated skin 8 weeks after radiation compared to unexpanded non-irradiated skin (FC = 8.1%, adj. *p*-value = 0.0003) (Figure 3A,B). Simultaneously, collagen content in unexpanded irradiated (71.7%) and expanded irradiated skin samples, regardless of which tissue expansion model was used (TE model = 73.4%, ADM model = 71.57%), was not significantly different. Similarly, we did not observe significant differences in collagen content in expanded irradiated skin (TE and ADM models) compared to non-irradiated samples. These results demonstrate that unexpanded skin is more susceptible to radiation-induced excessive collagen production than skin which underwent expansion prior to radiation. Notably, unlike the expanded non-irradiated skin, use of ADM in expanded skin followed by radiation did not protect against a radiation-induced increase in collagen deposition. 

### 2.4. Biological Cover Prevents Capsule formation in Expanded Non-Irradiated Skin

To evaluate the potential protective role of ADM in preventing capsule formation during tissue expansion, we carefully examined tissue around the tissue expanders after 2 and 10 weeks of expansion. IF staining of ACTA2 revealed a layer of ACTA2^+^ cells formed exclusively above the expander without an ADM cover at weeks 2 and 10 of expansion (Figure 3C). Radiation is known to accelerate fibrotic tissue formation [13], therefore we decided to verify if ADM also protects against capsule formation in expanded irradiated skin. IF staining for ACTA2 on expanded irradiated skin collected 8 weeks after radiation (10 weeks of expansion) showed numerous ACTA2^+^ cells on sections collected from both tested models, with and without an ADM cover (Figure 3C). However, capsules that developed above ADM depicted a less aligned and chaotic structure compared to capsules above the uncovered tissue expander. These results indicate that ADM covering the tissue expander limits the risk of mechanically induced capsule formation in non-irradiated expanded tissue. However, ADM does not fully prevent capsule formation in irradiated expanded skin, but rather alters capsule structure making it less stiff and perhaps less likely to contract. 

## 3. Discussion

Although tissue expansion has been used for many years in the clinical setting, minimal technological advancement has been introduced to this technique. In this study, we explored the potential beneficial effect of a biological cover on the dermal changes induced by mechanical forces during tissue expansion. To maintain strictly controlled conditions and ensure high similarity with human skin biology, the experimental procedures were executed on Yucatan minipigs [15,16]. We found that implanted ADM is well tolerated by a recipient and macrophages are involved in its incorporation into host tissue. Furthermore, our work revealed that use of ADM as a tissue expander cover limits excessive collagen production and the risk of capsule formation in non-irradiated skin.

Every biological scaffold evokes a host immune response. If the response is well tolerated, it leads to tissue remodeling and desired biomaterial incorporation [17,18]. Our study confirms previously published data, showing that ADM becomes an integral part of host tissue [19,20]. We demonstrated that macrophages play a crucial role in this process and their temporally recruitment to the region where ADM was adjacent to host tissue was necessary to restore the homeostatic state. The observed response resembles macrophage behavior during wound healing and regeneration, where these cells engage in restoring tissue homeostasis by extracellular matrix remodeling, and synthesis of cytokines and growth factors [21]. Furthermore, the evaluation of proinflammatory cytokine expression (*CCL2*, *TNFA*) confirmed that the use of a biological cover does not induce a prolonged inflammatory response. A brief increase in *CCL2* and *TNFA* expression was also observed in skin expanded without a biological cover. The significance of stretch-activated immune response for skin growth and regeneration has been shown previously on rats [22] and in porcine model [23,24]. Our results validate these findings and are in agreement with studies that emphasized the essential role of macrophages in successful incorporation of implanted biomaterials to yield optimal outcomes [17,25]. 

It has been shown that the dermis exposed to mechanical forces applied by the tissue expander becomes thinner [26,27]. While at an early stage of expansion we observed a greater decrease in the dermal thickness in skin expanded by the tissue expander with a biological cover, over time, this difference diminished. This result indicates that, in the long term, use of the biological cover in tissue expansion has no bearing on thinning of the dermis. 

Structural changes in collagen fiber orientation are correlated with strength of the tissue and its adaptability to multidirectional loads [28]. We noticed that tissue expansion interferes with and disturbs skin homeostasis, as presented by analysis of collagen fibers on Picrosirius red stained slides. Simultaneously, we determined that ADM partially mitigates dermal structural changes induced by expansion. We speculate that this might be explained by the changes in distribution and magnitude of mechanical forces applied by the tissue expander when ADM covers the tissue expander. The finite element analysis of skin growth and deformations will help to understand why tissue expansion with the biological cover causes less disruption than the standard procedure. 

Furthermore, we observed that the biological cover may prevent excessive collagen deposition by activated fibroblasts during tissue expansion [29,30]. Although a moderate increase in collagen content in response to tensile forces is associated with higher regenerative potential of skin [31], excessive collagen production results in increased skin stiffness and loss of elasticity [32]. Here, we presented that use of the tissue expander cover seems to reduce the risk of local overstimulation and helps to maintain balance between the pro-regenerative and the pro-fibrotic processes. We hypothesize that this protective effect of the biological cover may be partially explained by the changes in distribution of mechanical forces mediated by use of ADM.

Overactivation of the host inflammatory response often leads to the development of a capsule [33] or fibrous layer with excessive collagen production [34] around the prosthesis. In our study, we observed an increase in the presence of ACTA2^+^ cells around the expander in the TE model. ACTA2^+^ cells are highly specific for ɑ-smooth muscle actin (SMA), whose expression by myofibroblasts can increase due to exogenous mechanical forces and has been identified as the probable cause of capsular contracture in other studies [35,36]. Of the complications ascribed to prosthetic-based breast reconstruction, capsular contracture is a commonly reported endpoint and significantly influences patient satisfaction [37]. As such, preventative measures against capsule formation in expanded skin may minimize the progressive risk of capsular contracture and provide therapeutic benefit to the patient. 

It has been suggested that the implantation of a decellularized interface such as ADM between the prosthesis and surrounding tissue may block host inflammatory cells due to the absence of antigenic epitopes, prevent the development of a capsule, and lower the risk of capsular contracture [8,9,11,38,39,40,41]. In our study, we confirm the previous findings of a decrease in myofibroblasts in skin expanded with a biological cover [41]. However, in the setting of radiation, we demonstrated the added finding of decreased alignment of myofibroblasts in skin expanded with a biological cover compared to skin expanded without a cover. Differences in myofibroblast alignment have been previously implicated in wound healing and contracture, such that the more aligned the myofibroblasts, the more likely the capsule is to contract [35,42]. Although the use of a biological cover during tissue expansion does not appear to prevent capsule formation in irradiated skin, it may prevent pathological contracture of the capsule. 

## 4. Material and Methods

### 4.1. Ethics Statement

The animals were housed in animal facility (Center for Comparative Medicine, Northwestern University, Chicago, IL, USA) accredited by the Association for Assessment and Accreditation of Laboratory Animal Care International. All experiments were conducted according to the approved protocol number IS00010747 and in accordance with guidelines established by the Office of Laboratory Animal Welfare. 

### 4.2. Animal Model and Study Design

**Figure 4:**
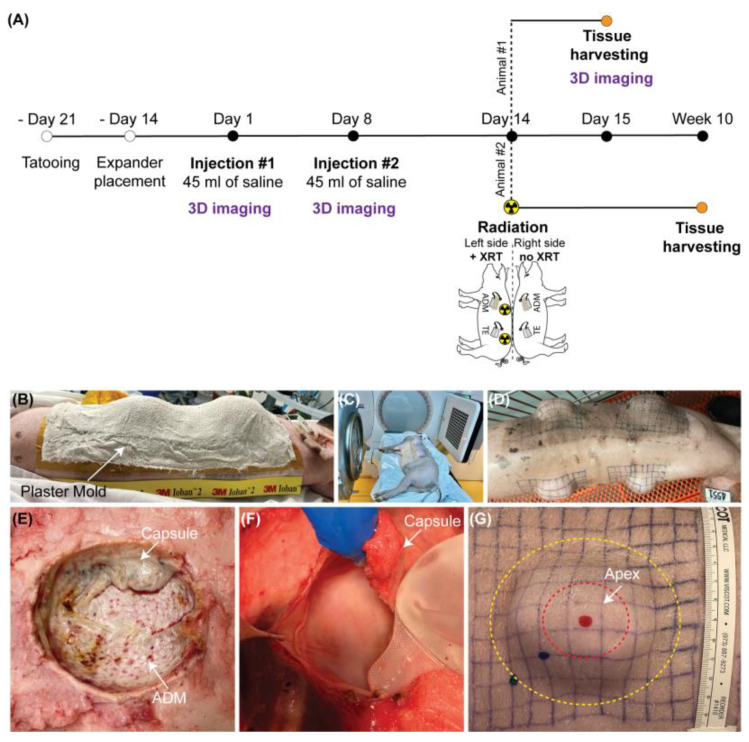
**Overview of the study design.** (**A**) Experimental timeline of the animal study. (**B**–**G**) Images presenting (**B**) utilization of a plaster mold of the intended radiation field, (**C**) pig radiation, (**D**) comparison between radiated and non-irradiated pig skin sites, (**E**) the underside of excised skin expanded with a tissue expander covered with acellular dermal matrix, (**F**) removal of the tissue expander encapsulated by connective tissue, (**G**) location of the apex of expanded skin, where skin biopsies were harvested from.

The tissue expansion procedures were performed on two 2-month-old female Yucatan minipigs (Premier BioSource) [43]. Both minipigs underwent tattooing of four 10 × 10 cm grids on the back and implantation of a 100 mL rectangular tissue expander (Mentor) below each grid. Half of the expanders were wrapped in a contoured sheet of acellular dermal matrix (ADM, FlexHD^®^ Pliable Acellular Hydrated dermis, MTF Biologics) and secured using absorbable sutures. After a two-week recovery, all expanders were inflated with two weekly fills of 45 mL. One week after the final inflation, the first pig was euthanized, and the second pig received unilateral single fraction radiation of 20 Gy. The second pig was subsequently euthanized 8 weeks later, on week 10 of expansion, which approximates the effects of radiation given to postmastectomy patients [44,45,46]. Contralateral expanded grids served as non-irradiated controls. Unexpanded non-irradiated skin was collected to establish a baseline for experimental conditions (Figure 4A–G). For each timepoint all tested conditions (control, tissue expansion without and with ADM in non-irradiated and irradiated skin) were performed simultaneously on the same animal to ensure that observed changes do not depend on individual predisposition of utilized animal and/or surgical skills of the surgeon.

On the day of euthanasia, the full-thickness punch biopsies were harvested from the apex of expanded skin and corresponding locations on the control grids. Three 4 mm biopsies were fixed in 10% formalin for 24 h at room temperature and embedded in paraffin blocks for histological and immunohistochemical analyses. Additional six 3 mm biopsies were preserved in Allprotect Tissue Reagent (Qiagen, Hilden, Germany) for RNA extraction.

### 4.3. Radiotherapy Protocol 

The radiotherapy protocol was established in collaboration with the Northwestern University Department of Radiation Oncology to mimic the human breast cancer treatment regimen. To avoid additional anesthetic events and unnecessary pig transportation, a plaster mold of the intended radiation field was created after the final fill (Figure 4B). The mold underwent CT simulation on a Philips Brilliance Big Bore 16 slice scanner to design a treatment plan. On the day of radiation, the anesthetized animal was positioned and immobilized in the same position as the CT simulation. Animal received unilateral single fraction radiation of 20 Gy [45,46] utilizing megavoltage photon beam energies (6–10 MV), arranged in two oblique gantry angles at a standard 100 cm source-to-axis distance on an Elekta Infinity linear accelerator with photon radiation (Figure 4C). In a manner akin to human breast cancer treatment, a 2 mm bolus was placed over the treatment field to minimize skin sparing properties of megavoltage photon beams. Beam shaping and avoidance of dose outside the intended target was accomplished by utilizing tangential beam arrangements and multi-leaf collimators. The radiation field was extended approximately 1–2 cm past the grid to establish a margin for dose build-up to reach the prescription dose. A field-in-field technique was utilized to make a more homogeneous dose distribution by creating additional blocking with multi-leaf collimators over the areas of higher dose for a small portion of beam on-time.

### 4.4. Masson Trichrome Staining 

Paraffin-embedded formalin-fixed skin sections (4 μm), placed on Superfrost Plus Microscope Slides (Fisher Scientific, Hampton, NH, USA), were stained with Masson Trichrome Staining Kit (Sigma-Aldrich, St. Louis, MO, USA) for histological evaluation, and mounted with Eco-Mount medium (Biocare Medical, Pacheco, CA, USA). The modifications introduced to the standard procedure of the manufacturer’s protocol are as follows: slides were incubated in Bouin’s solution at 56 °C for 1 h, stained with Working Weigert’s Iron Hematoxylin Solution (Sigma-Aldrich) for 30 min, incubated in Biebrich Scarlet-Acid Fuchsin for 10–15 min, differentiated in Phosphomolybdic-Phosphotungstic Acid Solution for 15 min, stained in Aniline Blue Solution for 10 min and differentiated in 1% acetic acid for 3–4 min. Stained slides were imaged on a Keyence BZ-X810 microscope. The quantitative analysis of collagen deposition was performed on images taken at 20× magnification, using ImageJ Fiji software v2.1.0/1.53c [47] and Colour Deconvolution plugin [48,49], which enables the separation of blue, red and green stains from an RGB image. The blue stain, representing collagen fibers, was used to calculate the percentage area of collagen deposition by adjusting the maximum threshold to highlight only the area where collagen was presented (blue hue). All images were analyzed using the average value of the maximum threshold calculated on control slides. A minimum 3 microscopic fields per section (n ≥ 9) and 3 non-consecutive sections per biopsy (n = 3) were analyzed. 

### 4.5. Immunofluorescence (IF) Staining

IF staining was performed on paraffin-embedded formalin-fixed cross sections as described previously [24]. Implemented modifications and used antibodies are listed below. Antigen retrieval was performed using the eBioscience™ IHC Antigen Retrieval Solution—Low pH (Invitrogen, Waltham, MA, USA) in the pressure cooker for 20 min. Next, slides were cooled at room temperature for 30 min and washed in PBS and PBST (0.1% Tween-20). The sections were incubated with the following primary antibodies: anti-CD68 (1:1000; MA513324, Invitrogen) and anti-iNOS (1:500; PA1036, Invitrogen) overnight at 4 °C. Signal was detected using fluorochrome-conjugated secondary antibodies: goat anti-mouse IgG Alexa Fluor Plus 488 (1:500; A11001, Invitrogen), donkey anti-rabbit Alexa Fluor Plus 546 (1:500; A10040, Invitrogen), and nuclei were counterstained using 1 μg/mL DAPI (Invitrogen). The specificity of the staining was tested by performing staining without the primary antibodies. 

### 4.6. Total RNA Extraction

Preserved tissue was submerged in an RLT buffer (Qiagen) with β-mercaptoethanol (1:100) and homogenized in 3 cycles for 10 min at 50,000 Hz in the cold room using TissueLyser LT homogenizer (Qiagen) paired with 7 mm stainless-steel beads (Qiagen). After each cycle, tissue lysate was examined to monitor the progress of homogenization. Next, tissue lysate was incubated with 200 µg of proteinase K in the water bath at 56 °C for 20 min, followed by RNA extraction using RNAeasy Mini Kit (Qiagen) with DNase I treatment (Qiagen) according to the manufacturer’s recommendations. Quality control was performed using 1% agarose gel. The concentration was measured on Nanodrop Spectrophotometers (DeNovix, Wilmington, DE, USA). 

### 4.7. Quantitative Real-Time PCR Analysis (qRT-PCR)

The qRT-PCR analysis was performed as described before [23] on QuantStudio 6 Flex Real-Time PCR Systems (Applied Biosystems, Waltham, MA, USA) using TaqMan Assay (Applied Biosystems), including commercially available TaqMan probes for each tested gene. The cycle quantification (Cq) values were calculated using the threshold cycle method and fold change of gene expression was calculated with the 2^−ΔΔCT^ method [50]. The results were normalized to the geometric mean calculated for 2 reference genes: *RLPL0* (Ribosomal Protein Lateral Stalk Subunit P0) and *RPS27A* (Ribosomal Protein S27a).

### 4.8. Measurement of the Dermal Thickness 

The dermal thickness was measured using ImageJ Fiji software on histological sections stained with Masson Trichrome (Sigma-Aldrich). Briefly, the surface area of the dermis was outlined using the Polygon tool found on ImageJ Fiji software and divided by the length of the bottom layer of the dermis to calculate the dermal thickness. The dermal thickness was analyzed on 1 microscopic field (images at 2× magnification) per each section (n ≥ 9). A minimum of 3 non-consecutive sections per biopsy (n = 3) were photographed and analyzed. 

### 4.9. Picrosirius Red Staining 

To visualize and evaluate collagen fiber orientation, slides were stained with picrosirius red solution using Picrosirius Red Stain Kit (Abcam, Cambridge, UK) according to the manufacturer’s recommendations. Stained slides were imaged on a Zeiss LSM880 confocal microscope using a 25× oil objective. The exposure time was identical between controls and experimental slides. CT-FIRE software [51] was used to analyze fiber angle, length, straightness, and width. A minimum of 3 microscopic fields per section (n ≥ 9) and 3 non-consecutive sections per biopsy (n = 3) were analyzed for each tested condition. 

## 5. Conclusions

Our study elucidates how use of the biological cover impacts structural changes in the dermis induced by mechanical forces during tissue expansion. We demonstrated that the biological cover is well tolerated by the recipient and macrophages are involved in its incorporation. We revealed that the biological cover may limit disturbance of dermal homeostasis induced by expansion, such as excessive collagen deposition, capsule formation, and disorganization of the dermal structure. However, the protective effect of the biological cover was not fully maintained in irradiated skin. Our work set the foundation for future studies on how to improve clinical outcomes in patients undergoing breast reconstruction.

### Limitation

The limiting factor of our study is the utilization of one specimen per tested timepoint. However, both experimental conditions, tissue expansion with, and without ADM, and corresponding control were performed simultaneously on the same animal, ensures that observed changes are not a result of inter-individual variations between animals. Although, the results presented in this paper demonstrate a potential beneficial effect of ADM on skin growth in tissue expansion, additional biological repeats are necessary to verify clinical significance of these findings. 

## Data Availability

All data generated or analyzed during this study are included in this article.

## Abbreviations

ADM, acellular dermal matrix; SD, standard deviation; FDA; IF, immunofluorescence; qRT-PCR, quantitative real-time PCR.
